# Rituximab as possible therapy in TNF inhibitor-induced IgA vasculitis with severe renal involvement

**DOI:** 10.1186/s12882-023-03439-0

**Published:** 2023-12-20

**Authors:** Agnieszka Przygocka, Gian Marco Berti, Anita Campus, Francesco Tondolo, Gisella Vischini, Benedetta Fabbrizio, Gaetano La Manna, Olga Baraldi

**Affiliations:** 1 Department of Medical and Surgical Sciences (DIMEC), Alma Mater Studiorum - University of Bologna, Bologna, Italy, Via Giuseppe Massarenti 9, Bologna, Italy; 2 Nephrology, Dialysis and Kidney Transplant Unit, IRCCS Azienda Ospedaliero-Universitaria di Bologna, Via Giuseppe Massarenti 9, Bologna, Italy; 3 Pathology Unit, IRCCS Azienda Ospedaliero-Universitaria di Bologna, Via Giuseppe Massarenti 9, Bologna, Italy

**Keywords:** Drug-induced vasculitis, IgA vasculitis, Acute kidney injury, Tumor necrosis factor inhibitors, Golimumab, Adalimumab, Rituximab

## Abstract

**Background:**

We observe the increasing use of tumor necrosis factor (TNF) inhibitors in patients affected by chronic inflammatory diseases. These drugs provide good control of symptoms, contributing to significant improvement in the quality of life in individuals with high disease burden. On the other hand, along with their wider use and longer follow-up periods the number of reports regarding their adverse effects is also increasing. The reported complications include drug-induced vasculitis with possible kidney involvement. In the literature we can distinguish more frequently described ANCA-associated vasculitis and more rarely occurring immunoglobulin A vasculitis. Although uncommon, such complications may present with potentially life-threatening vital organ dysfunction; therefore, adequate monitoring and effective therapy are necessary.

**Case presentation:**

We report two cases of TNF inhibitor-induced vasculitis with severe acute worsening of renal function and significant proteinuria. The first patient was receiving golimumab therapy for ankylosing spondylitis and the second patient was treated with adalimumab for psoriasis and psoriatic arthritis. In the second case dialysis treatment was necessary and the patient presented recurrence of vasculitis after rechallenge with adalimumab. Both patients underwent renal biopsy which showed findings compatible with drug-induced IgA vasculitis and both were treated successfully with corticosteroids and rituximab.

**Conclusions:**

To the best of our knowledge this is the first report of rituximab use in drug-induced IgA vasculitis with renal involvement. Combination of corticosteroids and rituximab can be an effective therapy in case of vasculitis with kidney failure and a preferable option for selected patients with drug-induced IgA vasculitis compared to cyclophosphamide. More studies are necessary to establish suitable short- and long-term treatment. Given the rarity of this disorder, case reports and case series can provide practical guidance until additional studies become available.

## Key points

Drug-induced vasculitis is a rare complication of anti-TNF therapy and can present with systemic (including renal) involvement.

Careful monitoring during anti-TNF therapy, including serum creatinine and proteinuria (baseline and periodic follow-up exams), is essential to allow prompt diagnosis and treatment.

TNF inhibitors can cause AAV and IgAV; kidney biopsy is a useful tool for confirming the diagnosis and introducing appropriate treatment.

No guidelines regarding treatment are currently available. In the case of systemic involvement corticosteroid therapy and a second immunosuppressive agent (cyclophosphamide, rituximab) can be considered; in the case of IgAV rituximab may be preferable in selected patients.

Maintenance therapy might not be necessary provided that the offending agent is withdrawn.

## Background

Over the years tumor necrosis factor (TNF) inhibitor therapy has proven to be an effective treatment for chronic inflammatory diseases with a good safety profile. However, with the increase in patients receiving anti-TNF therapy and longer follow-up periods the number of reports addressing its adverse effects is rising. One of the possible complications is drug-induced vasculitis and the kidney is one of the most commonly affected organs in patients with systemic involvement [1, 2]. Currently there are no guidelines regarding the management of this disorder. We present two case reports of patients who developed drug-induced IgA vasculitis (IgAV) during TNF inhibitor therapy and discuss the treatment options.

## Case report 1

A 68-year-old Caucasian woman was admitted to our ward because of acute kidney injury (AKI). Her past medical history was remarkable for ankylosing spondylitis diagnosed 14 years ago, treated with different lines of treatment (methotrexate, hydroxychloroquine, etanercept, secukinumab and infliximab), at the time of admission on therapy with golimumab since 6 years. Prior to hospitalization her renal function was within normal limits.

She presented with petechiae affecting the upper and lower limbs which appeared after golimumab injection. After 4 days the skin lesions were followed by low grade fever and macrohematuria. Her history was negative for recent infections and she denied any new medication use. The laboratory analysis revealed AKI with serum creatinine (sCr) of 2.5 mg/dL (baseline 0.6 mg/dL) and estimated glomerular filtration rate (eGFR) of 21 mL/min (baseline 98 mL/min). The urine examination showed subnephrotic proteinuria and hematuria. Immunological exams were negative for antineutrophil cytoplasmic antibodies (ANCA), antinuclear antibodies (ANA), anti-glomerular basal membrane antibodies (anti-GBM), anti-phospholipase A2 receptor antibodies (anti-PLA2R) and anti-thrombospondin-2 antibodies (anti-THBS2). The complement level was normal. The infection workup excluded hepatitis B, C and tuberculosis. Renal ultrasound findings were within normal limits. Thoracic and abdominal imaging did not reveal signs of malignancy. Treatment with golimumab was suspended. The patient underwent a kidney biopsy which showed immunoglobulin A (IgA) dominant proliferative extracapillary glomerulonephritis. Skin biopsy of the purpuric lesions revealed leukocytoclastic vasculitis (immunofluorescence staining was not available).

**Fig. 1:**
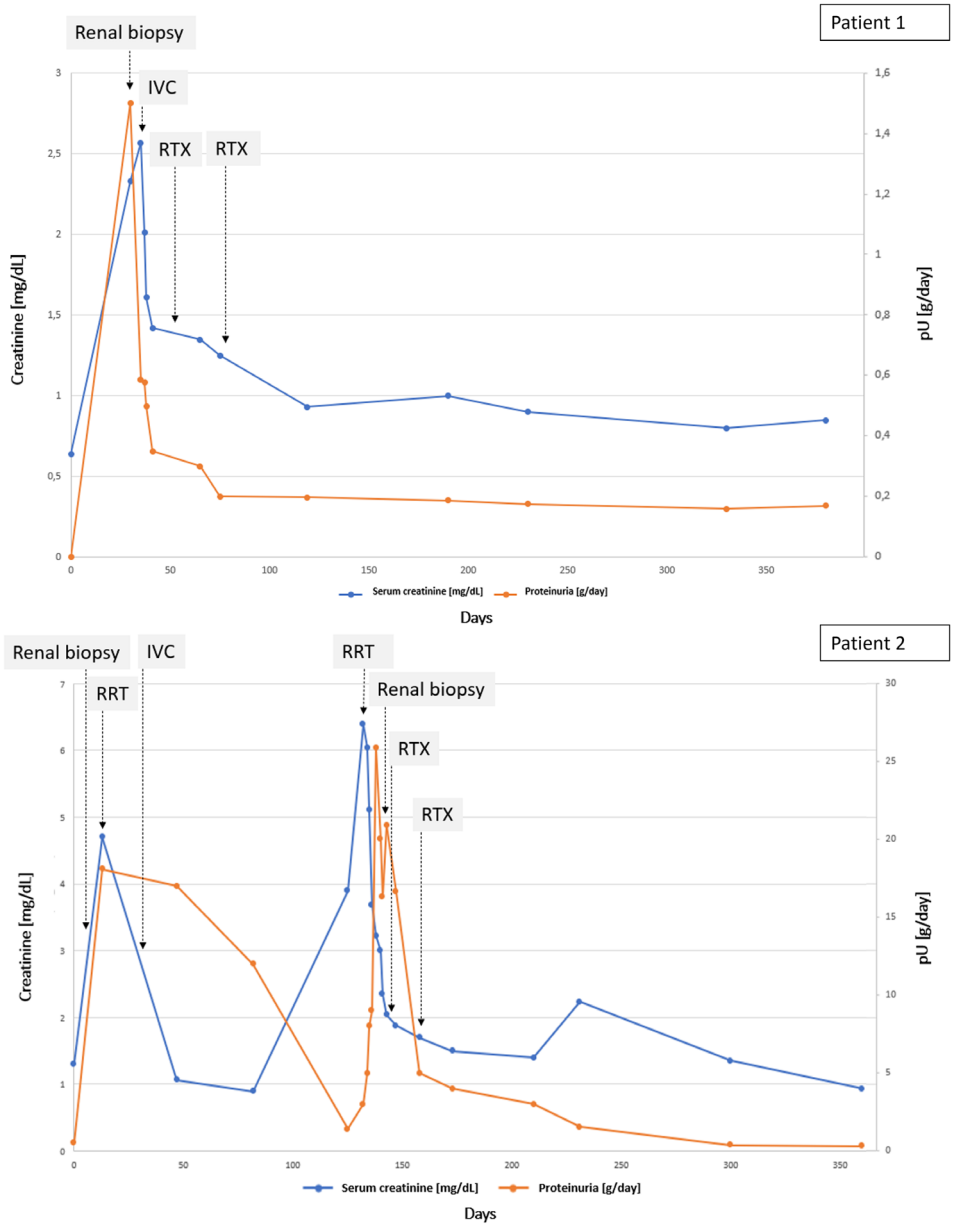
Timeline of the serum creatinine and proteinuria of patient 1 and patient 2. IVC: intravenous corticosteroids; pU: proteinuria; RRT: renal replacement therapy; RTX: rituximab

Considering the pathological and clinical findings the patient received immunosuppressive treatment with corticosteroids (methylprednisolone 125 mg followed by dose tapering) and rituximab (2 infusions of 1 g, two weeks apart) with subsequent rapid improvement of kidney function. Follow-up exams at 1 year confirmed remission (sCr 0,84 mg/dL, eGFR 71 mL/min and urinalysis within normal values) (Fig. 1).

## Case report 2

A 49-year-old Caucasian man was hospitalized in our department for hypertension, widespread edema and weight gain of 17 kg over a week. The patient had a 20-year history of psoriasis and psoriatic arthritis, at the time on therapy with adalimumab and the symptoms presented after the last drug administration. No recent infectious events or new drug use was reported and he did not experience joint pain or gastrointestinal disorders. He was previously treated with corticosteroids, cyclosporin and methotrexate and was receiving therapy with adalimumab since one year at the time of hospitalization. In the last five years he presented microscopic hematuria and proteinuria upon urine examination, but a nephrological evaluation was not performed. His serum renal function indices were normal.

The laboratory exams revealed severe renal dysfunction (sCr 3.56 mg/dL, eGFR 19 mL/min) with nephrotic-range proteinuria (18 g/day) and microhematuria. The rest of the blood analysis results were within the normal range. His immunological screening exams were negative. Hepatitis B, C and tuberculosis infections were excluded. Renal ultrasound and chest X-ray did not reveal any abnormalities. Adalimumab was discontinued and in consideration of severe renal failure with oliguria urgent hemodialysis treatment was started after central venous catheter (CVC) placement.

Renal biopsy was performed and revealed proliferative extracapillary glomerulonephritis with predominant IgA deposits and massive acute tubular necrosis. Renal-limited IgAV was suspected; thus, intravenous steroid therapy was started (methylprednisolone 1 g for three consecutive days and subsequent dose tapering) with complete recovery of renal function. Further immunosuppressive treatment was not performed because of CVC-related infection occurred during hospitalization. At the time of discharge sCr was 0.9 mg/dL (eGFR 101 mL/min).

**Fig. 2:**
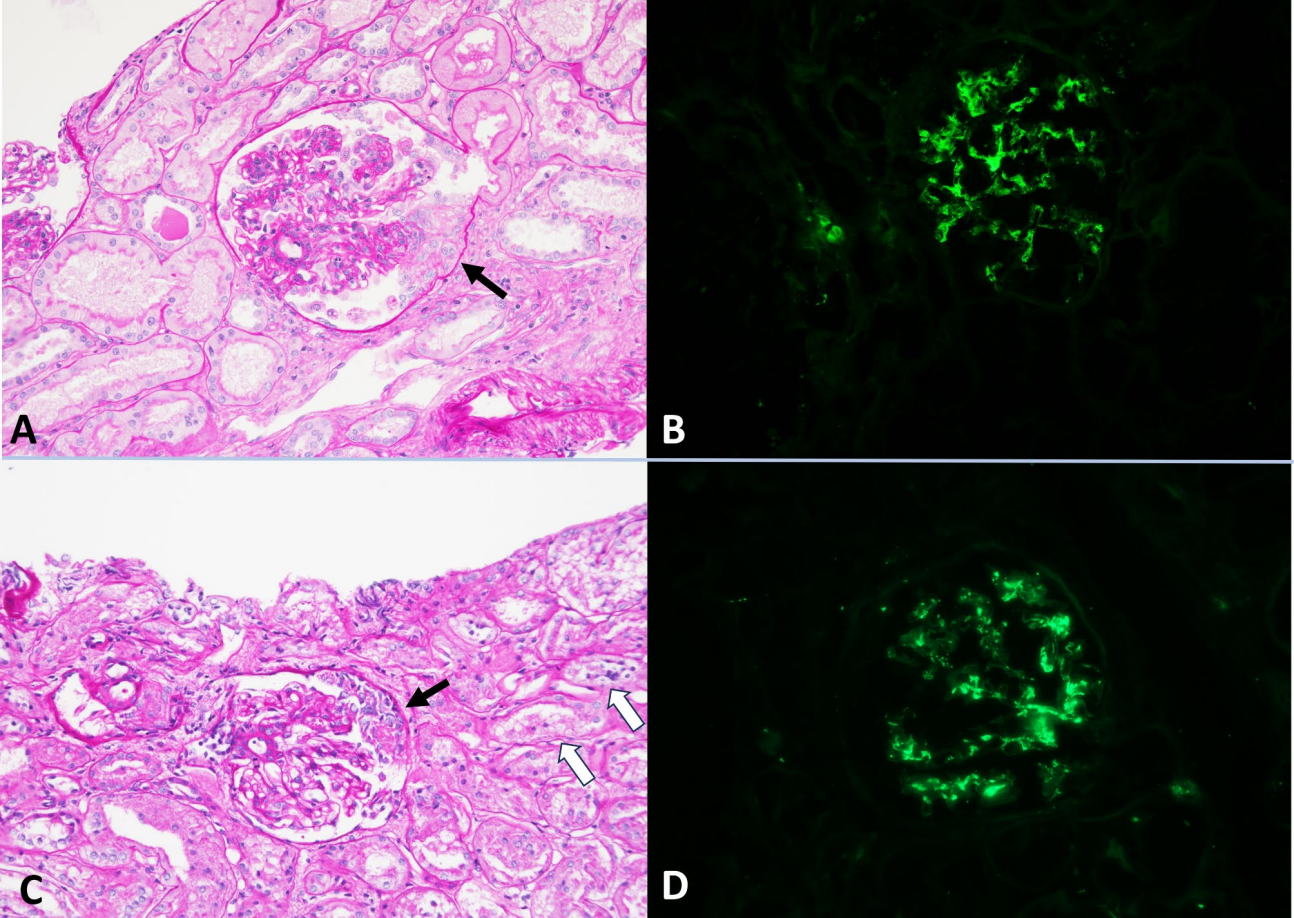
Kidney biopsy findings in patient 1 **(A, B)** and patient 2 **(C, D)**. Light microscopy image showing a glomerulus with thickened capillary loops and the presence of a cellular crescent (arrow) (**A**, periodic acid-Schiff stain x20). Immunofluorescence staining revealed immunoglobulin A dominant deposits in the mesangium (**B**, immunofluorescence x20). Acute tubular necrosis (white arrows), glomerulus with cellular crescent (black arrow) and fibrinoid necrosis (**C**, periodic acid-Schiff stain x20). Presence of immunoglobulin A mesangial deposits (**D**, immunofluorescence x20)

A few months later the patient experienced a relapse of the psoriatic lesions and adalimumab was reintroduced. After 5 administrations of adalimumab he developed severe fluid retention, hypertension and oliguria; therefore, he was referred to the Emergency Department where laboratory tests confirmed another episode of AKI (sCreat 3.9 mg/dL) with nephrotic-range proteinuria (14 g/day). During the following hospitalization one hemodialysis session was performed because of the persistence of oligoanuria. Adalimumab was suspended indefinitely and a second kidney biopsy was carried out, which showed recurrence of glomerulonephritis with IgA deposits. Relapse of IgAV was postulated; hence, the patient received immunosuppressive therapy with corticosteroids (methylprednisolone 1 g with subsequent tapering) and rituximab (2 infusions of 1 g, two weeks apart). We observed rapid kidney function improvement and the patient did not require further dialysis treatments. A good response to therapy was confirmed at follow-up at 1 year (sCreat 0.96 mg/dL, eGFR 92 ml/min and urinalysis within the normal range) (Fig. 2)

## Discussion

Various medications are associated with drug-induced IgAV. A large pharmacovigilance study by Rasmussen et al. identified three main groups as possible culprit agents: vaccines, antibiotics (in particular beta-lactamines, fluoroquinolones and macrolides) and immunomodulatory drugs (including TNF inhibitors) [3].

IgAV (formerly known as Henoch-Schönlein purpura) is characterized by the accumulation of IgA1-dominant immune complexes in small vessels [4]. Its pathogenesis remains unclear and it is most likely multifactorial. In a novel multi-hit model Heineke et al. described the possible role of IgA anti-endothelial cell antibodies which bind to small vessels causing neutrophil activation. IgA-activated neutrophils release TNF-alpha which is presumed to stimulate endothelial cells leading to further inflammation [5]. It is hypothesized that both genetic factors (susceptibility genes) and environmental triggers (infections, drugs) are involved [3]. In genetically predisposed individuals aberrant glycosylation of IgA1 may lead to the production and accumulation of immune complexes [5]. On the other hand, exposure to medications can stimulate autoantibody production and provoke neutrophil apoptosis, causing glomerular damage [6–8]. In the case of anti-TNF agents the inhibition of TNF-alpha causes cytokine imbalance by switching the cell response from T-helper type 1 to type 2, with a consequent prevalence of proinflammatory cytokines such as interleukin-1 (IL-1), IL-6 and IL-17/23 [9, 10]. Moreover, some studies have suggested the formation of an anti-TNF/TNF immune complex that can deposit in small vessels [3, 11]. From the nephrological point of view anti-TNF agents have been linked to ANCA-related glomerulonephritis and IgAV in some cases and rarely they can be associated with podocytopathies [3, 12].

According to the International Chapel Hill Consensus Conference Nomenclature of Vasculitides, drug-induced IgAV is classified as vasculitis associated with probable etiology [4], as its cause cannot be proven with certainty. The incidence of the disease is difficult to estimate and in the few available studies on drug-induced IgAV it has not been reported. In our center the two described patients are the only drug-associated IgAV cases identified in recent years. Its diagnosis is assumed by temporal association of clinical findings with drug administration and by exclusion of other causes. The comprehensive medication history of the patient should be evaluated, including drug use for at least six months before manifestation of the symptoms [1]. The most common clinical presentations are palpable purpura, arthralgia and/or arthritis, gastrointestinal disorders and, more rarely, renal involvement with glomerulonephritis [3]. Rasmussen et al. reported glomerulonephritis in one-third of patients with drug-induced IgAV [3]. In the setting of TNF inhibitors, a study by Ramos-Casals et al. revealed kidney involvement in 13.5% of patients with vasculitis induced by anti-TNF therapy (although it included all types of vasculitis and not only IgAV) [13]. It is often challenging to determine causality in the case of suspected drug-induced IgAV but improvement of vasculitis symptoms after drug withdrawal can suggest its role in the development of this disease. Renal biopsy is a useful tool for confirming the diagnosis, assessing disease severity and determining appropriate treatment [1]. A review of the available literature of reported cases of IgAV with kidney involvement during TNF inhibitor therapy [14–18] is presented in Table 1.

**Table 1:** Literature review of reported cases of IgA vasculitis with renal involvement during TNF inhibitor therapy. ANA: anti-nuclear antibodies; CS: corticosteroids; CYC: cyclophosphamide; MMF: mycophenolate mofetil; N/A: not available

Author, year	Number of cases	TNF inhibitor	Serology positivity	Withdrawal of TNF inhibitor	Treatment	Outcome
Duffy et al., 2006 [14]	1	Etanercept	ANA	Yes	CS, CYC	Good response
Sokumbi et al., 2012 [15]	1	Infliximab	N/A	Yes	CS, MMF	No response
Rolle et al., 2013 [16]	1	Etanercept	ANA, anti-histone	Yes	CS	Good response
Hokama et al., 2019 [17]	1	Adalimumab	Negative tests	Yes	CS	Good response
Fukuda et al., 2023 [18]	1	Infliximab	N/A	Yes	CS	Good response

Given the rarity of drug-induced vasculitis, there is currently no standard approach to its therapy and data on drug-induced IgAV treatment are even more scarce. It is recommended to promptly withdraw the offending drug which is usually sufficient for patients with mild disease. Immunosuppressive therapy should be started only in case of vital organ involvement. The available data generally extrapolate treatment recommendations from primary vasculitis (including AAV) guidelines to drug-induced vasculitis [19]. In severe forms of drug-induced vasculitis, the reports in the literature suggest treatment with corticosteroids and the addition of a second immunosuppressive agent if necessary. Prednisone at 1 mg/kg should be used for 4 to 8 weeks with subsequent tapering over 6 to 12 months; intravenous methylprednisolone of 1000 mg per day for 3 consecutive days may be considered for rapidly progressive glomerulonephritis [1, 6, 20]. In the case of severe worsening of renal function or vital pulmonary involvement (respiratory failure, alveolar hemorrhage), a second immunosuppressive agent should be added. In such a setting, cyclophosphamide and rituximab are proposed [6, 9], and the majority of reports of severe drug-induced vasculitis describe treatment with cyclophosphamide as a second agent [21–25]. Maintenance therapy may not be necessary provided that the offending drug is discontinued [1]. Rechallenge should be avoided because it can cause relapse of vasculitis, as in our second patient. Considering the possible class effect, if TNF inhibitor-induced vasculitis occurs, an alternative class agent may be indicated; careful monitoring is needed since cases of vasculitis recurrence after a change in the anti-TNF agent have been reported [26].

Cyclophosphamide is an alkylating drug with good efficacy in vasculitis with renal involvement (with most evidence regarding AAV) but concomitant significant toxicity. The main concerns regard the risk of bladder cancer, leukemia and gonadal toxicity, particularly in the case of prolonged exposure [27–29]. A promising alternative agent is rituximab, a chimeric monoclonal antibody directed against the CD20 antigen of B cells. Randomized controlled trials (RAVE [30] and RITUXVAS [31]) have shown the noninferiority of rituximab to cyclophosphamide for remission induction in AAV. A study of AAV patients by van Daalen et al. [32] showed a 4.61-fold greater long-term malignancy risk in the cyclophosphamide group and the risk comparable with that in the general population in the rituximab group. The successful use of rituximab in the setting of drug-induced vasculitis with renal impairment has been reported in several publications [33–36], including reports by Florez et al. and Funada et al. who described cases of ANCA-associated glomerulonephritis induced by an anti-TNF agent (etanercept and certolizumab pegol, respectively) treated with rituximab with a sustained response [33, 34].

In primary IgAV, the addition of cyclophosphamide to corticosteroids in adults in a randomized controlled trial by Pillebout et al. [37] did not show a benefit compared to steroid therapy alone. However, survival at 12 months insignificantly differed (79% in the corticosteroid group, 96% in the cyclophosphamide and corticosteroid group); moreover, as noted by the authors, the small sample size (due to recruitment challenge) did not allow to draw definitive conclusions. On the other hand, although evidence on rituximab use in IgAV is limited, this treatment has shown good efficacy in both adult and pediatric populations (as rescue therapy and a corticosteroid-sparing regimen but also as the only immunomodulatory treatment) [38–41]. In a multicenter observational study of 22 patients with adult-onset IgAV, Maritati et al. [38] reported that rituximab induced disease remission and allowed corticosteroid tapering. Similar results were described in a case series by Fenoglio et al. [39]. The mechanism of rituximab in IgAV is not completely understood; it is hypothesized that it may decrease tissue-infiltrating B cells and IgA-producing plasma cells, and debilitate other functions of B cells [38]. There are currently no reports on the use of rituximab in drug-induced IgAV but our experience suggests that it could be a promising therapeutic option in this setting. However, rituximab should be used with caution in frail patients who present a higher risk of severe infections during the course of immunosuppressive therapy which can be associated with treatment failure [42, 43].

Overall, the prognosis in drug-induced IgAV appears to be good as long as the offending drug is withdrawn. In the study by Rasmussen et al. [3] 65.2% of patients with drug-induced IgAV did not require vasculitis-specific treatment, 53.9% presented complete recovery, 13% had sequelae and no deaths were reported. The recovery rate was better in the pediatric population than in adults, consistent with the data in the literature on primary IgAV [38, 44]. 1.7% of patients had persistent chronic renal failure at follow up. Meanwhile, in primary IgAV the risk of developing chronic kidney disease is estimated to be approximately 18% in adults [44]. However, in the study by Rasmussen et al. no separate outcome analysis was performed for patients with renal involvement [3]. Since primary IgAV with kidney involvement tends to have a worse prognosis [38, 44], evaluating whether such a trend is also present in patients with drug-induced IgAV would be useful in further studies.

Chronic inflammatory diseases may be associated with other autoimmune disorders. The coexistence of IgA nephropathy with ankylosing spondylitis [45–48] has been reported relatively frequently, as has psoriasis and psoriatic arthritis [49–51]. In recent years there has also been growing evidence on the association of IgAV with these diseases [52–54]. Some studies suggest that IgA nephropathy, ankylosing spondylitis and psoriasis share common pathophysiological mechanisms such as increased serum IgA levels and alterations in cytokine pathways [55–57]. IgA nephropathy and IgAV are considered related disorders [5]; therefore, it is plausible that there may also be a correlation between IgAV and these inflammatory diseases. Nevertheless, such associations appear rare and in our two patients drug-related etiology seems more plausible given the absence of vasculitis symptoms in long-standing chronic inflammatory disease before starting anti-TNF therapy and because of the very good response after drug withdrawal and immunosuppressive therapy, with no need for specific maintenance treatment. We believe that in both patients the presence of ankylosing spondylitis and psoriasis can be additional risk factors for the development of vasculitis but are not necessarily the most important causes. In the first patient the appearance of suspected drug-induced IgA vasculitis after 6 years of therapy with golimumab is unusual, but cases of vasculitis after long-term use of TNF inhibitors have been reported. Sokumbi et al. [15] described cases of vasculitis after 60 and 72 months of therapy with anti-TNF agents, although only cases with cutaneous involvement were reported after such a long period of therapy. After excluding other causes in our patients we believe that this is the most likely diagnosis, although it cannot be proven with certainty.

In the second patient it is possible that underlying but undiagnosed IgA nephropathy was present (considering the presence of hematuria and proteinuria in the previous years); however, the strong correlation of symptoms with adalimumab administration, recurrence of vasculitis at drug rechallenge and complete resolution of heavy proteinuria after drug discontinuation suggest that the use of an anti-TNF agent was an important component of the disease cause.

In light of the available data, we believe that rituximab can be a valid treatment option for severe cases of vasculitis with renal involvement requiring intensive immunosuppressive therapy, also in the setting of drug-induced vasculitis. It can be a safe alternative to cyclophosphamide, particularly in young patients, considering fertility preservation and lower malignancy risk, and a preferable option in selected cases of IgAV. Currently there are few data in the literature on the use of rituximab in IgAV as primitive vasculitis and there are no data on its use in drug-induced IgAV. Our experience suggests that this approach could also be a valid therapy in the setting of drug-related IgAV. The two reported patients showed a good response to treatment with complete recovery of renal function, which was confirmed at follow-up at 1 year. Randomized trials are needed to confirm the efficacy of rituximab in IgAV but considering the rarity of this disorder, such studies are unlikely to be available in the near future. Case reports and case series can provide practical guidance until additional studies are performed.

To the best of our knowledge, this is also the first report to describing a patient with features of both glomerulonephritis and leukocytoclastic vasculitis after the administration of golimumab.

## Conclusion

In conclusion, careful monitoring, including kidney function assessment, is necessary during anti-TNF therapy as prompt diagnosis and treatment in patients with vasculitis complications can allow satisfactory renal recovery. We recommend regular examination of renal function markers (serum creatinine, eGFR, proteinuria) in patients receiving therapy with anti-TNF agents. Combination of corticosteroids and rituximab can be an effective therapy in case of drug-induced vasculitis with severe renal involvement, in the setting of both ANCA-associated and IgA vasculitis. However, more studies are needed to establish appropriate treatment.

## Data Availability

The data generated and analyzed in this case are presented within the manuscript.

## Abbreviations

AAV: ANCA associated vasculitis
AKI: Acute kidney injury
ANA: Antinuclear antibodies
ANCA: Antineutrophil cytoplasmic antibodies
Anti-GBM: Anti-glomerular basal membrane antibodies
Anti-PLA2R: Anti-phospholipase A2 receptor antibodies
Anti-THBS2: Anti-thrombospondin-2 antibodies
CVC: Central venous catheter
eGFR: Estimated glomerular filtration rate
IgAV: Immunoglobulin A vasculitis
IL: Interleukin
sCr: Serum creatinine
TNF: Tumor necrosis factor
